# Pre-Weaning Growth Hormone Treatment Ameliorates Bone Marrow Macrophage Inflammation in Adult Male Rat Offspring following Maternal Undernutrition

**DOI:** 10.1371/journal.pone.0068262

**Published:** 2013-07-02

**Authors:** Clare M. Reynolds, Minglan Li, Clint Gray, Mark H. Vickers

**Affiliations:** Liggins Institute and Gravida, National Research Centre for Growth and Development, University of Auckland, Auckland, New Zealand; State University of Rio de Janeiro, Biomedical Center, Institute of Biology, Brazil

## Abstract

Maternal undernutrition (UN) is associated with the development of obesity and metabolic complications in adult offspring. While the role of inflammation in obesity and related comorbidities has been well established, there is little evidence regarding the effects of maternal UN-induced programming on immune function in male adult offspring. This study examines the effects growth hormone (GH), which is known to induce anti-inflammatory effects, on maternal UN-induced bone marrow macrophage (BMM) function in adult male offspring. Sprague-Dawley rats were assigned to chow (C) or UN (50% ad libitum; UN) diet throughout gestation. Male C and UN pups received saline (CS/UNS) or GH (2.5 µg/g/d; CGH/UNGH) from day 3–21. Bone marrow hematopoietic cells were differentiated to a macrophage phenotype in the presence of M-CSF (50 ng/ml). Differentiated bone marrow macrophages (BMM) were stimulated with LPS (100 ng/ml) for 6 h. UNS-derived BMM had significantly increased secretion and expression of IL-1β and IL-6 following LPS stimulation. This was accompanied by increased expression of IL-1R1, IL-6R and TLR4. Pre-weaning GH treatment reversed this pro-inflammatory phenotype. Furthermore UNGH displayed increased expression of markers of alternative (M2) macrophage activation, mannose receptor and PPARγ. This study demonstrates that fetal UN exposure primes hematopoietic immune cells to a more potent pro-inflammatory phenotype with heightened cytokine secretion and receptor expression. Furthermore these cells are pre-disposed to pro-inflammatory M1 macrophage phenotype which has wide-reaching and important effects in terms of obesity and metabolic disease.

## Introduction

There is considerable evidence that exposure to adverse fetal and early life nutritional and environmental stressors result in increased risk of adult disease [1]. The predominant focus of the “developmental origins of health and disease” (DOHaD) hypothesis has largely centred around perturbations in metabolic homeostasis [2], [3]. However, recent studies have provided insight into alteration of immune function in response to gestational nutritional imbalances [4]. Indeed maternal nutrition is associated with placental inflammation and aberrant immune activity which may alter nutritional set points established throughout gestation and the neonatal period enhancing the risk not only for obesity-induced metabolic dysfunction but also chronic inflammatory conditions later in life [5]. Furthermore there is significant evidence that maternal undernutrition (UN) has detrimental effects on the development of both primary and secondary lymphoid organs [6].

The innate immune system is the first line of defense against invading organisms. However, dysfunctional activation of these innate immune cells contributes to the pathogenesis of both metabolic and chronic inflammatory disorders [7]. Macrophages represent a critical part of this system. They originate in the bone marrow as monocytes and once differentiated are distributed throughout most body tissues. In addition to their role in inflammation, macrophages are key mediators of metabolic function and tissue remodeling. Given their multifunctional nature, macrophages display significant phenotypic plasticity. Classically activated (M1) macrophages typically produce high levels of pro-inflammatory cytokines (interleukin (IL)-12, IL-1β, Tumor necrosis factor (TNF)α and IL-6) while alternatively activated (M2) macrophages are characterized by anti-inflammatory IL-10 and IL-4 [8]. Macrophages originate early in embryonic development and as such may be vulnerable to maternal programming [9]. Despite this, the role of developmental programming on long-term immune function has not been comprehensively investigated. Furthermore viable therapeutic treatments to address immunological disparities in relation to maternal UN-induced programming remain unexplored.

Several studies have reported beneficial effects of growth hormone (GH) on parameters of metabolic function in the offspring of UN mothers [10], [11]. Indeed, recent evidence from this group has determined that pre-weaning GH treatment ameliorates hypertension in these animals [12]. Traditionally GH has been implicated in the regulation of key components of lipid and glucose homeostasis however growing evidence has established GH as a contributor in the development of immune function and there is evidence from both human and rodent models that GH treatment induces anti-inflammatory effects [13], [14]. The GH receptor (GHR) is expressed on wide variety of innate and adaptive immune cells, including bone marrow-derived cells [15], therefore we speculated that pre-weaning GH treatment may influence the development of immune function.

The present study investigates the impact maternal UN on the bone marrow-derived macrophage (BMM) cytokine secretion, immune receptor expression and markers relevant to macrophage polarization. Furthermore we assess the contribution of early life GH administration on the amelioration of this immunophenotype. This data reveals that BMM from UN mothers exhibit a pre-primed pro-inflammatory phenotype which can be rescued following pre-weaning GH treatment.

## Materials and Methods

### Animal Experiment and Intervention

Virgin Sprague-Dawley rats were time-mated using a rat oestrous cycle monitor prior to introduction of males. Following mating, females were housed individually with free access to water. All rats were maintained in the same room with a constant temperature of 25°C and a 12 h light:dark cycle. Animals were randomly assigned to either dietary group a) standard diet (C, Diet 2018, Harlan Teklad, Oxon, UK) available *ad libitum* throughout pregnancy or group b) undernutrition (UN); 50% of *ad libitum* diet throughout pregnancy. After birth (day 2) litters were adjusted to eight pups per litter to ensure adequate and standardized nutrition until weaning. Pups were assigned to either saline (S) control group or growth hormone (GH) treated group (bovine GH (bGH, Cyanamid; 2.5 µg/g) and injected subcutaneously daily from day 3–21. After weaning (day 21) offspring were housed 2 per cage and fed the standard diet for the remainder of the study. Only male offspring were used in the present study to avoid the confounds of estrus. Body weights were measured every 3 days throughout the study. Adult offspring body composition was measured by dual energy X-ray absorptiometry (DEXA) on day 150 (Lunar Prodigy, GE, Waltham, USA). On day 160, rats were fasted overnight and killed by sodium pentobarbitone (60 mg/kg, IP) anesthesia followed by decapitation. All animal work was approved by the Animal Ethics Committee of the University of Auckland.

### Materials

Primers, probes and TaqMan Universal Mastermix were purchased from Applied Biosystems (ABI, CA). All other reagents were purchased from Sigma Aldrich unless otherwise stated.

### Bone Marrow Macrophage (BMM) Isolation and Culture

BMM were prepared by culturing bone marrow cells obtained from the femurs and tibia of rats in DMEM medium supplemented with macrophage-colony stimulating factor (rat recombinant M-CSF; 50 ng/ml; Sigma-Aldrich). Cells were isolated from CS, CGH, UNS and UNGH groups fed a chow diet to determine the effects of maternal programming on immune cell function. Following 7 d culture cells were stimulated for 6 h with lipopolysaccharide (LPS) (100 ng/ml) for cytokine and RNA analysis. Supernatants were stored for cytokine analysis by ELISA and cells were harvested for RNA analysis.

### Cytokine Analysis

Supernatants from the BMM activation experiments were analysed for IL-1β, IL-6, TNFα and IL-10 by commercial Quantikine ELISA kits (R&D Systems; Abingdon, UK), according to the manufacturer’s instructions.

### Gene Expression Analysis

RNA was extracted from BMM using TRI-Reagent and stored at −80°C. Single-stranded cDNA was prepared using High-Capacity cDNA Archive Kit (Applied Biosystems, Warrington, UK). mRNA expression was quantified by real-time PCR (RT-PCR) on an ABI 7700 Sequence Detection System (Perkin-Elmer Applied Biosystems). To control for between-sample variability, mRNA levels were normalized to the geometric mean of cyclophilinA and hypoxanthine phosphoribosyltransferase (HPRT) for each sample by subtracting the Ct of controls from the Ct for the gene of interest producing a ΔCt value. The ΔCt for each treatment sample was compared to the mean ΔCt for control samples (CS) using the relative quantification 2-(ΔΔCt) method to determine fold-change.

### Statistics

Three way-Analysis of variance (ANOVA) was used to determine significant differences in BMM between conditions with maternal diet, pre-weaning GH treatment and LPS stimulation as factors. Two-way ANOVA was carried out on plasma data to determine differences between conditions with maternal diet and pre-weaning GH treatment as factors. Data are presented as means ± SEM. When this indicated significance (p<0.05), post-hoc Bonferroni test analysis was used to determine which conditions were significantly different from each other. All statistical analysis was performed using SPSS version 14.0 for Windows (SPSS Inc., Chicago, IL).

## Results

### Phenotypic Characteristics

**Figure 1:**
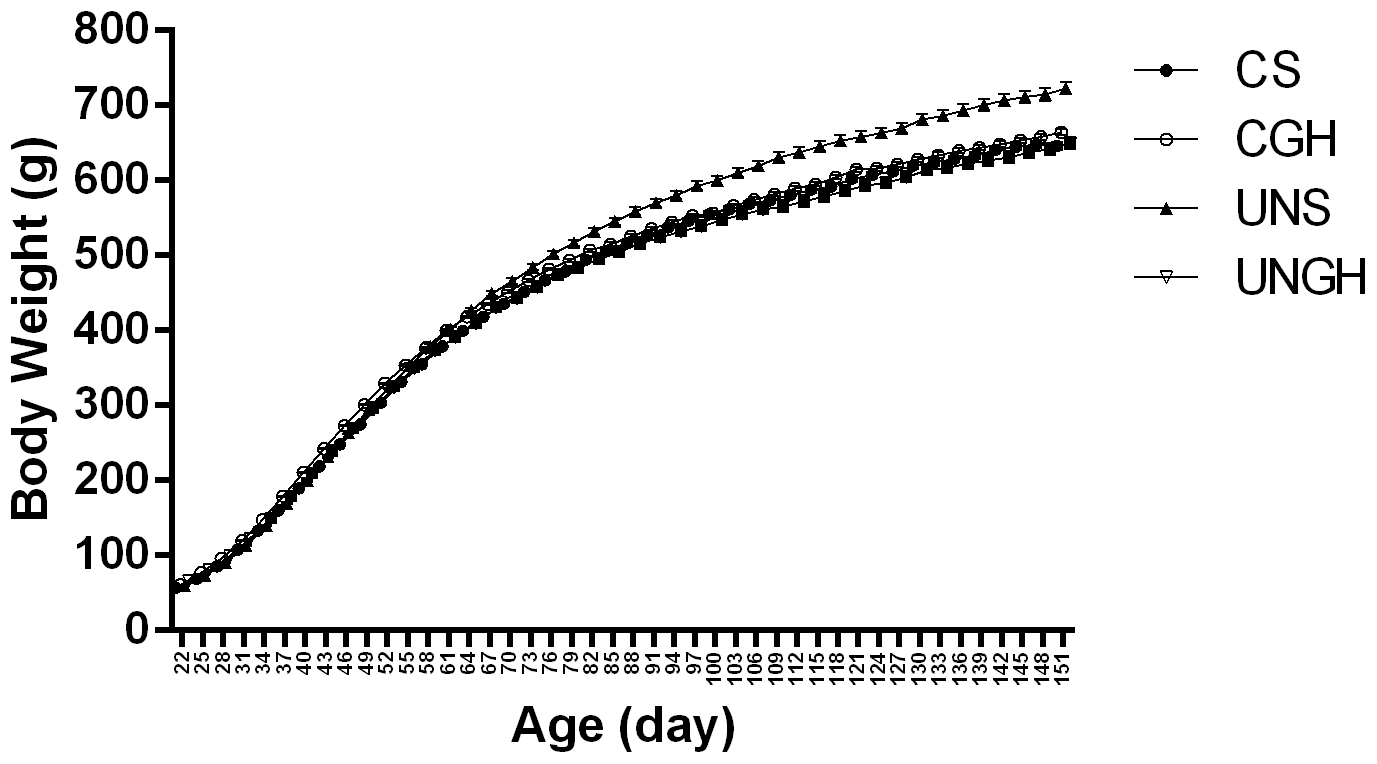
Growth curves. Body weight was assessed throughout the study. Cumulative body weights were calculated and values were expressed as mean ± SEM.

In line with previously published data, male offspring of UN mothers had increased body weight compared to CS, CGH and UNGH groups (Figure 1). This was accompanied by increases in fat mass and circulating pro-inflammatory cytokines, IL-1β, IL-6 and TNFα in UNS compared to CS, CGH and UNGH groups (Table 1). Furthermore, insulin resistance, macrophage infiltration and increased inflammation in adipose tissue of UN offspring was observed (data not shown). These effects were ameliorated in UNGH offspring.

**Table 1:** Plasma profile.

	Body Weight (g)	Fat Mass (%)	TNFα (pg/ml)	IL-1β (pg/ml)	IL-6 (pg/ml)
**CS**	648±18	31.6±1.6	14.48±3.26	60.69±11.7	82.14±4.12
**CGH**	650±17	36.8±2.8	23.79±4.04	103.1±12.3	149.62±15.4
**UNS**	711±30*	42.3±2.9**	25.93±4.15*	205.9±30.2*	131.6±14.5**
**UNGH**	643±13^+^	35.71±1.7^+^	2.560±0.61^+++^	50.12±20.1+	62.64±4.6^+++^
*Maternal diet (MD)*	*p<0.05*	*p<0.05*	*NS*	*p<0.05*	*NS*
*GHtx (GH)*	*NS*	*NS*	*NS*	*p<0.01*	*NS*
*MDxGH*	*NS*	*p<0.05*	*p<0.001*	*p<0.001*	*p<0.001*

Plasma was isolated from CS, CGH, UNS, UNGH groups and metabolic markers were analysed enzymatically. (*p<0.05, **p<0.01 w.r.t CS; ^+^p<0.05, ^++^p<0.01; ^+++^p<0.001 w.r.t UNS, n = 4). Values were expressed as mean ± SEM. Two-way ANOVA indicates significance in relation to Maternal diet (MD), GH treatment (GHtx) and the interaction between maternal diet and GH treatment (MD×GH). NS denotes non-significance.

### BMM Cytokine Profile

**Figure 2:**
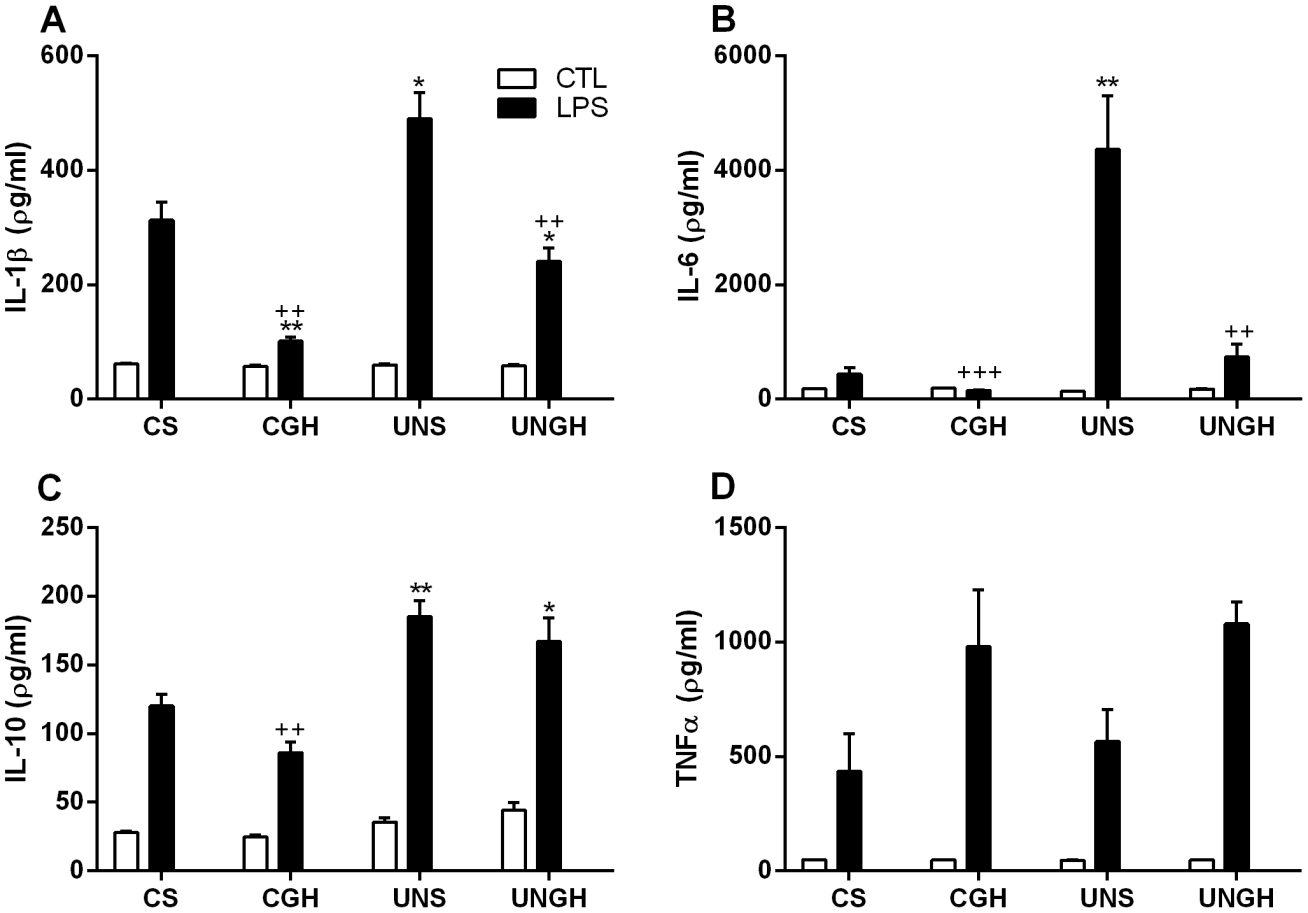
Bone marrow was isolated from CS, CGH, UNS and UNGH male adult rats. Cells were cultured for 7 d in the presence of M-CSF (50 ng/ml) to differentiate to BMM. Once differentiated BMM were stimulated for 6 h with LPS (100 ng/ml). Cytokine analysis for (A) IL-1β, (B) IL-6, (C) IL-10 and (D) TNFα was carried out using Quantikine ELISA (*p<0.05, **p<0.01, ***p<0.001 w.r.t CS; ^+^p<0.05, ^++^p<0.01, ^+++^p<0.001 w.r.t UNS, n = 4). Values were expressed as mean ± SEM.

Following stimulation with LPS, there were significantly enhanced IL-1β and IL-6 concentrations in UNS compared to CS, CGH and UNGH-derived BMM (Figure 2A&B). Interestingly there were increased IL-10 concentrations in UNS and UNGH compared to CS BMM however there was no difference between UNS and UNGH derived BMM (Figure 2C). There was no difference in TNFα concentrations between groups (Figure 2D).

### BMM Inflammatory Gene Expression

**Figure 3:**
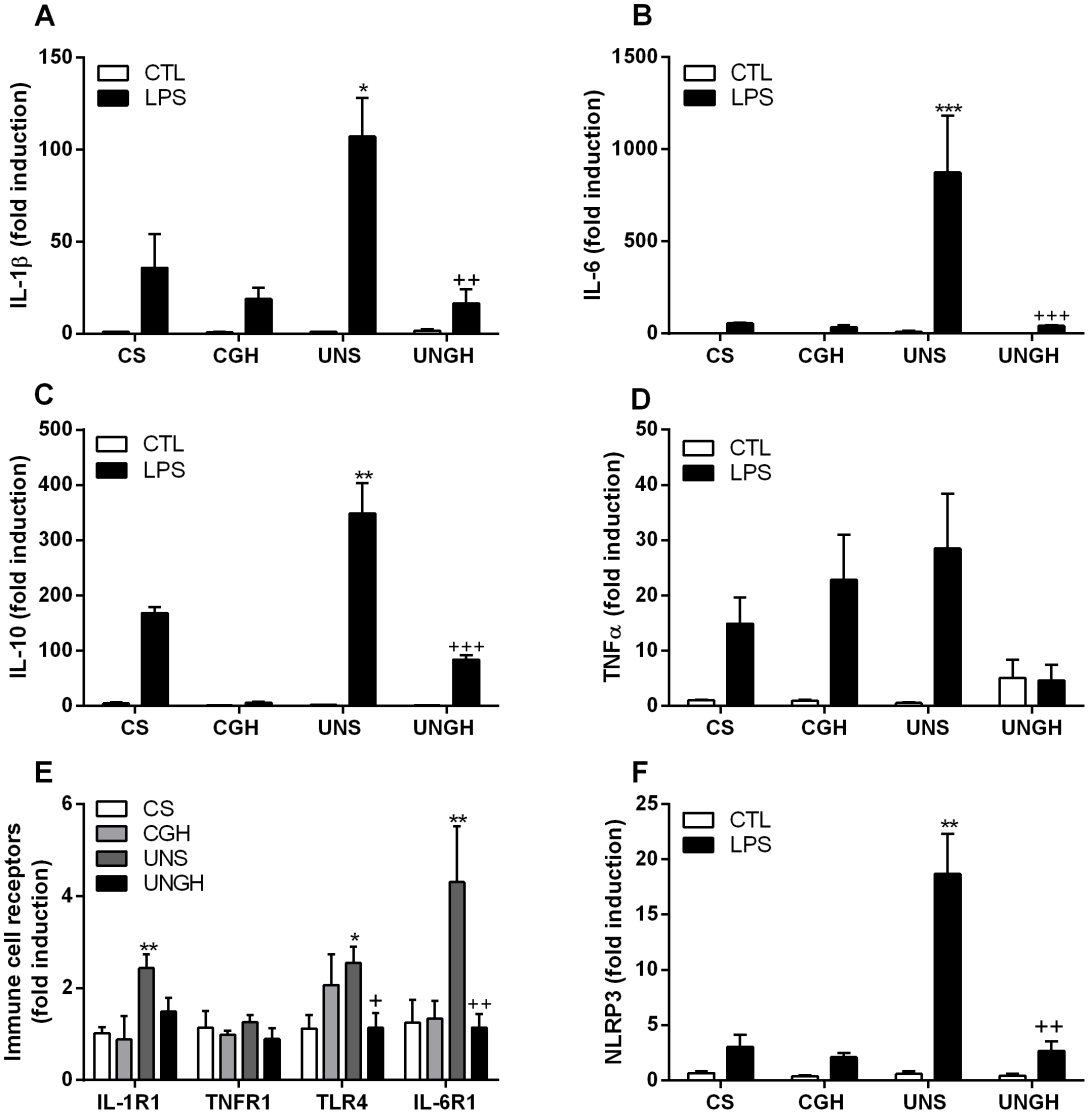
BMM cytokine and cytokine receptor gene expression. Bone marrow was isolated from CS, CGH, UNS and UNGH male adult rats. Cells were cultured for 7 d in the presence of M-CSF (50 ng/ml) to differentiate to BMM. Once differentiated BMM were stimulated for 6 h with LPS (100 ng/ml). Gene expression of (A) IL-1β, (B) IL-6, (C) IL-10, (D) TNFα, (E) immune cell receptors and (F) NLRP3 were analysed by RT-PCR. (*p<0.05, **p<0.01, ***p<0.001 w.r.t CS; ^+^p<0.05, ^++^p<0.01, ^+++^p<0.001 w.r.t UNS, n = 4). Values were expressed as mean ± SEM.

IL-1β, IL-6 and IL-10 mRNA expression was significantly increased in UNS compared to CS, CGH and UNGH-derived BMM (Figure 3A–C). However there were no significant differences in TNFα expression between groups (Figure 3D). IL-1R1, IL-6R1 and toll-like receptor (TLR)4 were significantly increased in UNS compared to CS, CGH and UNGH groups, TNFR1 remained unchanged between groups (Figure 3E). Furthermore NLRP3, a critical component of the IL-1β processing machinery, was significantly up-regulated in UNS compared to CS, CGH and UNGH groups (Figure 3F).

### BMM Polarization Potential

**Figure 4:**
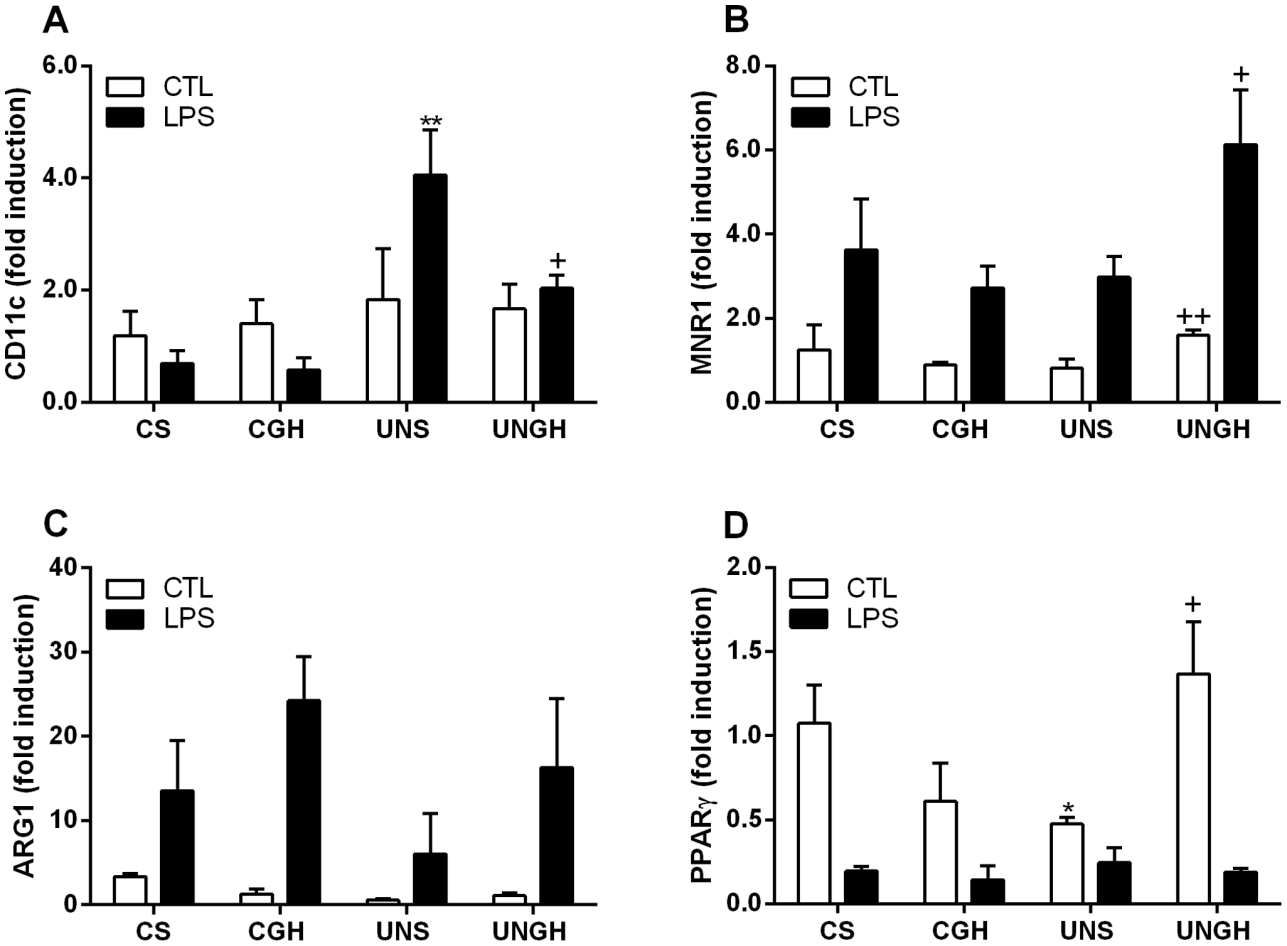
Macrophage polarisation gene expression. Bone marrow was isolated from CS, CGH, UNS and UNGH male adult rats. Cells were cultured for 7 d in the presence of M-CSF (50 ng/ml) to differentiate to BMM. Once differentiated, BMM were stimulated for 6 h with LPS (100 ng/ml). Gene expression of (A) CD11c, (B) MNR1, (C) ARG1 and (D) PPARγ were analysed by RT-PCR. (*p<0.05, **p<0.01 w.r.t CS; ^+^p<0.05, ^++^p<0.01 w.r.t UNS, n = 4). Values were expressed as mean ± SEM.

In response to LPS stimulation UNS-derived BMM demonstrated significantly increased CD11c expression compared to CS, CGH and UNGH (Figure 4A). Arginase (ARG)1 and mannose receptor (MNR)1, markers indicative of M2 macrophages were assessed. While there was no significant difference in MNR1 gene expression between CS, CGH and UNS-derived BMM, there was significantly increased expression in the UNGH compared to UNS-derived BMM. There was no significant change in ARG1 gene expression between groups. Furthermore expression of peroxisome proliferator-activated receptor (PPAR)γ, a key transcription factor regulating polarisation to M2 phenotype, was attenuated in the UNS BMM compared to CS, CGH and UNGH groups (Figure 4D).

## Discussion

There is growing interest in the role of developmental programming in chronic adult onset conditions characterised by low-grade inflammation such as cardiovascular disease and type 2 diabetes [16]. Indeed, recent work by this group has demonstrated significant obesity and metabolic dysfunction in male offspring of UN mothers with beneficial effects of pre-weaning GH treatment on cardiovascular status and endothelial dysfunction [12]. However, evidence regarding the specific role of inflammatory processes with regards to maternal UN is lacking. As the immune system is continuously developing throughout gestation and lactation we speculated that exposure to maternal UN would influence the immunophenotype of bone marrow macrophages (BMM) in adult male offspring. Indeed, BMM from UN mothers have heightened secretion and expression of pro-inflammatory cytokines and their receptors which is reversed in animals which received pre-weaning GH treatment.

Evidence from human studies has indicated that the immune system begins to develop at an early embryonic stage. The fetal liver is the most significant contributor to the early immune system, however as it begins to develop metabolic capabilities, the bone marrow takes over as the primary source of hematopoietic stem cells [17]. While a rudimentary immune system is present at birth, the immune system does not fully mature until later in life and is therefore susceptible to programming events from gestation to the post-weaning period [6]. Indeed the current study demonstrates that maternal UN skews BMM to a more immunogenic phenotype.

Similar to previous studies conducted by this group and others, the current study demonstrates significantly increased body weight and adiposity in male adult offspring of UN mothers [18], [19]. These adverse effects on body composition are reversed following pre-weaning GH treatment. Given the low-grade pro-inflammatory state associated with obesity, we analysed systemic cytokine concentrations. UNS offspring had significantly increased TNFα, IL-1β and IL-6. These cytokines are integral to the pathogenesis of the metabolic syndrome and cardiovascular disease and represent a link between the immune system and metabolic dysfunction [20], [21]. Despite this, the contribution of inflammatory processes in developmental programming of metabolic disease has not been comprehensively investigated and there are conflicting observations in regards to systemic inflammation. None the less given the increased circulating cytokine concentrations in UNS offspring we sought to determine potential cellular origins of this pro-inflammatory phenotype. Several studies have demonstrated the impact of dietary intervention on the immunophenotype of BMM and dendritic cells [22]. Given their relative plasticity we speculated that these progenitor cells may be altered by maternal UN and may influence programming events leading to obesity and metabolic dysfunction. BMM represent a major culprit in the initiation of metabolic inflammation and contribute substantially to the systemic cytokine pool [23]. As such we conducted our study on macrophages differentiated from bone marrow hematopoietic cells.

Macrophages originate from pluripotent stem cells in the bone marrow, differentiate into monocytes in the blood and infiltrate body tissues as macrophages. Their versatile nature contributes to regulation of inflammatory processes, maintenance of cellular homeostasis and even embryonic growth [24]. Over the last decade the role of macrophages and their secretory products in metabolic dysfunction has been well established. [21]. In the current study, BMM isolated from UNS offspring have a significantly enhanced immunophenotype evidenced by secretion of large concentrations of IL-β and IL-6 accompanied by increased gene expression of TLR4, IL-1R1 and IL-6R. TLR4 plays a fundamental role in the innate immune system. It is activated in response to bacterial LPS, however given its propensity to also bind saturated fatty acids, it has been ascribed a role in the development of diet-induced metabolic dysfunction and obesity [25]. It is widely expressed on macrophages and activation initiates an inflammatory cascade which culminates in activation of nuclear factor kappa B (NF-κB), considered the master regulator of inflammatory processes.

Both IL-1β and IL-6 are known to be regulated in this manner and have been implicated in the pathogenesis of IR and progression to overt type-2 diabetes. Knock out models suggest that abolition of IL-1β or its receptor IL-1R1 partially protects against obesity-induced metabolic disease [21]. Furthermore, these knock out models display reduced macrophage infiltration into insulin sensitive organs along with reduced cytokine secretion from BMM indicating a potential role for IL-1β in maternal UN-mediated obesity and associated co-morbidities. Maternal UN is associated with reduced circulating GH concentrations in neonates; we speculate that this reduction may have an adverse impact on the developing immune system leading to heightened inflammatory potential leading to the observed adverse effects later in life. Interestingly IL-1β concentrations were normalised by pre-weaning GH administration. Several studies have demonstrated that GH treatment reduces IL-1β concentrations *in vitro* and *in vivo* [14], [26]. In addition NLRP3, a key component of the inflammasome complex which regulates the release of mature IL-1β is increased in UNS offspring BMM and restored with GH treatment. Previous studies have demonstrated the importance of this protein and emerging evidence has implicated inflammasome activation in the initiation of metabolic disease in response to diet and obesity [27]. Furthermore, NLRP3 has been shown to be altered in BMM in response to high-fat diet demonstrating its susceptibility to environmental alterations [28]. IL-1β is a key cytokine and dysregulation can contribute to disorders ranging from metabolic dysfunction to schizophrenia and rheumatoid arthritis [29]. Therefore evidence of UNS-mediated alterations of IL-1β and IL-1R1 in BMM may be important in understanding the mechanisms surrounding a range of conditions know to be influenced by developmental programming. This study also provides evidence of increased IL-6 and IL-6R concentrations in BMM from UNS offspring and a reversal of this phenotype in response to pre-weaning GH treatment. This is unsurprising as IL-1β stimulates IL-6 production through activation of p38 mitogen-activated protein kinase (MAPK) and signal transducer and activator of transcription (STAT)5 [30]. Like IL-1β, IL-6 has a key role in a wide variety of metabolic, neurological and inflammatory conditions and evidence of its up-regulation in response to UNS has far-reaching implications for developmentally programmed conditions.

Macrophages display significant plasticity in relation to their environment. There are two main classes of macrophage, classically activated (M1) macrophages are induced by TLR4 agonists such as saturated fatty acids or lipopolysaccharide (LPS), typically express the cell surface marker CD11c and have a pro-inflammatory cytokine profile; alternatively activated (M2) macrophages which produce anti-inflammatory cytokines such as IL-10 and are generally involved in the resolution of inflammation [31]. Given the enhanced pro-inflammatory profile of UNS-derived BMM we speculated that these cells were skewed towards an M1 phenotype and restoration of an M2 phenotype in response to pre-weaning GH treatment. We therefore examined expression of CD11c, a key determinant of M1 macrophage differentiation. As expected this was significantly enhanced in UNS-derived BMM in response to LPS. Furthermore we assessed markers such as IL-10, arginase 1 and mannose receptor which typify an M2 phenotype. While arginase-1 and mannose receptor expression were unchanged in UNS-derived BMM compared to control cells, there was a significant increase in UNGH-derived BMM both in non-treated and LPS stimulated cells. Interestingly there was no accompanying increase in IL-10 secretion or expression, indeed UNS-derived BMM demonstrated increased concentrations. This may reflect the role of IL-10 in counter-regulation of pro-inflammatory cytokine concentrations. PPARγ is an essential regulator of glucose and fatty acid metabolism. However it is clear that PPARγ is highly expressed in macrophage populations and acts to suppress pro-inflammatory mediators such as IL-1β [32]. Furthermore it has been shown to play a role in macrophage polarization, influencing a switch from M1 to M2 with studies demonstrating that inhibition of PPARγ in myeloid cells prevents polarization to an M2 phenotype predisposing to diet-induced obesity, insulin resistance and decreased fatty acid utilization [33]. PPARγ gene expression was significantly down-regulated in UNS-derived BMM, providing a mechanistic insight into the pro-inflammatory M1 phenotype observed in these cells. In line with other observations in this study, PPARγ expression was normalised in UNGH-derived BMM which may explain polarisation to a more favourable inflammatory phenotype.

### Conclusion

The role of inflammation and immune cell function in terms of maternal UN has not been comprehensively examined. Reports are inconsistent and many focus on circulating immune mediators and the adaptive immune system. This study examines the effects of maternal UN on a bone marrow-derived model of innate immune function. Enhancement of cell surface immune receptors coupled with increased cytokine secretion indicates a pre-primed pro-inflammatory innate immune in these animals. We further confirm that pre-weaning GH treatment reverses these programmed effects. However the role of GH in metabolic disease and inflammation is controversial as direct treatment with GH is known to induce insulin resistance and can promote inflammation [34]. Importantly the current study does not look at the direct effects of GH in adult offspring rather the early role of GH in directing the nature of immune responses and prevention of UNS-mediated adverse programming effects. Given that bone marrow progenitors have a high turnover rate it is likely they the alterations leading to this adverse phenotype are driven by epigenetic mechanisms, however further investigations are required to fully elucidate this. As inflammatory parameters are strongly associated with developmentally programmed disease states such as obesity and metabolic syndrome the implications of this study are wide-reaching and important in terms of developing realistic therapeutic strategies.
